# The Newfoundland and Labrador Bariatric Surgery Cohort Study: Rational and Study Protocol

**DOI:** 10.1186/s12913-016-1869-5

**Published:** 2016-10-28

**Authors:** Laurie K. Twells, Deborah M. Gregory, William K. Midodzi, Carla Dillon, Christopher S. Kovacs, Don MacDonald, Kendra K. Lester, David Pace, Chris Smith, Darrell Boone, Raleen Murphy

**Affiliations:** 1 School of Pharmacy, Memorial University, Health Sciences Centre, 300 Prince Philip Drive, St. John’s, NL A1B 3 V6 Canada; 2 Faculty of Medicine, Memorial University, Health Sciences Centre, 300 Prince Philip Drive, St. John’s, NL A1B 3 V6 Canada; 3 Eastern Health, Health Sciences Centre, 300 Prince Philip Drive, St. John’s, NL A1B 3 V6 Canada; 4 Research and Evaluation, Newfoundland and Labrador Centre for Health Information, 70 O’Leary Avenue, St. John’s, NL A1B 2C7 Canada

**Keywords:** Obesity, Bariatric surgery, Laparoscopic sleeve gastrectomy, Health outcomes, Canada

## Abstract

**Background:**

In Canada, there has been a disproportionate increase in adults with Class II (BMI 35.0–39.9 kg/m^2^) or Class III obesity (BMI ≥ 40 kg/m^2^) affecting 9 % of Canadians with increases projected. Individuals affected by severe obesity (BMI ≥ 35) are at increased risk of high blood pressure, cardiovascular disease, diabetes, cancer, impaired quality of life, and premature mortality. Bariatric surgery is the most effective treatment for severe obesity. Laparoscopic sleeve gastrectomy (LSG), a relatively new type of bariatric surgery, is growing in popularity as a treatment. The global prevalence of LSG increased from 0 to 37.0 % between 2003 and 2013. In Canada and the US, between 2011 and 2013, the number of LSG surgeries increased by 244 % and LSG now comprises 43 % of all bariatric surgeries. Since 2011, Eastern Health, the largest regional health authority in Newfoundland and Labrador (NL), Canada has performed approximately 100 LSG surgeries annually.

**Methods:**

A population-based prospective cohort study with pre and post surgical assessments at 1, 3, 6, 12, 18, 24 months and annually thereafter of patients undergoing LSG. This study will report on short - to mid-term (2–4 years) outcomes. Patients (*n* = 200) followed by the Provincial Bariatric Surgery Program between 19 and 70 years of age, with a BMI between 35.0 and 39.9 kg/m^2^ and an obesity-related comorbidity or with a BMI ≥ 40 kg/m^2^ are enrolled. The study is assessing the following outcomes: 1) complications of surgery including impact on nutritional status 2) weight loss/regain 3) improvement/resolution of comorbid conditions and a reduction in prescribed medications 4) patient reported outcomes using validated quality of life tools, and 5) impact of surgery on health services use and costs. We hypothesize a low complication rate, a marked reduction in weight, improvement/resolution of comorbid conditions, a reduction in related medications, improvement in quality of life, and a decrease in direct healthcare use and costs and indirect costs compared to pre-surgery.

**Discussion:**

Limited data on the impact of LSG as a stand-alone procedure on a number of outcomes exist. The findings from this study will help to inform evidence-based practice, clinical decision-making, and the development of health policy.

## Background

Obesity, most often defined using body mass index or BMI (kg/m^2^), is classified in categories that are reflective of the increasing health risk associated with excess body weight (Class I: BMI 30.0–34.9 kg/m^2^, Class II BMI 35.0–39.9 kg/m^2^, Class III BMI ≥ 40.0 kg/m^2^) [1, 2]. Severe obesity, herein defined as Class II and III, is associated with much higher levels of morbidity, increased demands on the health care system, and premature death [3–5]. It increases the risks of developing high blood pressure, cardiovascular disease and type 2 diabetes [3], significantly impairs quality of life [6], and shortens life expectancy [5].

Over the last three decades, the prevalence of obesity has increased in many countries [7]. In Canada, obesity currently affects 25 % of adults with severe obesity increasing over 400 % since 1985 and now affecting nearly 10 % of the population or approximately 1,000,000 Canadians [8, 9]. The estimated annual economic burden of obesity evidenced by direct costs related to hospitalizations, medication use, physician and emergency room visits, and indirect costs related to reduced work productivity and increased absenteeism, is now estimated to be between $4.6 billion and $7.1 billion [10, 11].

### Bariatric surgery

Severe obesity is difficult to treat. Treatments that include changes in diet, exercise, behavioral modification/counselling, medical management or pharmacotherapy have demonstrated limited effectiveness as successful treatments for weight loss or long-term weight control. On average these interventions result in a modest weight loss of 5–10 % of initial body weight [12–14].

Bariatric surgery, the most effective treatment for those with severe obesity, offers significant and sustained weight loss, improvement in comorbid conditions and quality of life, and reduces the risk of death [15–23]. Canadian guidelines exist on the surgical treatment of adult obesity. Surgery is indicated in medically refractory patients with a BMI ≥ 35 kg/m^2^ plus obesity-related comorbidity condition (e.g., hypertension, diabetes) or a BMI ≥ 40 kg/m^2^ [3].

There are several different types of bariatric surgery and all involve an alteration of the digestive system in either a restrictive, malabsorptive, or combination restrictive/malabsorptive capacity [3, 20–23]. Restrictive type bariatric procedures impose a physical limitation on the amount of food that can be consumed by reducing gastric volume. The most common procedures include (a) laparoscopic sleeve gastrectomy (LSG) where the greater curvature and fundus of the stomach are surgically resected leaving an elongated tube or stomach ‘sleeve’ and (b) adjustable gastric banding (AGB) where the proximal stomach is encircled with an adjustable band that is progressively inflated to create a small restrictive gastric pouch. Malabsorptive procedures restrict nutrient and calorie absorption in the small intestine and include the biliopancreatic diversion/duodenal switch (BPD/DS). Other bariatric surgeries use a combination of restriction and malabsorption to promote weight reduction, and of these the most commonly performed is the gold standard roux-en-y gastric bypass (RYGB) which results in a highly restrictive gastric pouch coupled with a diversion of the upper small intestine [21].

In 2003, 146,301 bariatric procedures were performed worldwide with the majority (103,000) performed in the United States and Canada. The types of procedures comprised of 85 % RYGB, 9 % AGB, and 4.5 % BPD/DS [24]. Since 2003, a significant shift has occurred in the type of procedure being performed. In 2013, the total number of worldwide procedures increased to 468,609 (with 154,276 performed in US/Canada) and was comprised of 45.0 % RYGB, 37.0 % LSG, 10.0 % AGB, and 1.5 % BPD/DS. Similar to the worldwide shift in type of procedure being performed, in Canada and the US, between 2011 and 2013, the number of LSG surgeries increased by 244 % and now comprises 43 % of all bariatric surgeries [25].

These changes reflect the exponential growth of LSG both worldwide and in the United States and Canada [25]. Over 90 % of bariatric surgeries are performed laparoscopically [21]. This minimally invasive approach reduces surgical risk, hospital stay, and recovery time compared to open techniques and has contributed to the increasing popularity of this approach [26].

### Bariatric surgery outcomes

A recently published systematic review and meta-analysis by Chang et al. [27] on the effectiveness and risks of bariatric surgery provides an update on studies published between 2003 and 2012 comparing surgical and non-surgical treatments for severe obesity. This review includes 164 studies (*n* = 161,756): 37 randomized controlled trials (RCT) and 127 observational studies (OBS). Just over half of the studies, 54 % (*n* = 91), provided follow-up data on patients for ≥ 2 years. The mean age (years) and BMI (kg/m^2^) of patients was 44.6 and 45.6, respectively and almost 80 % were women. Average pre-surgery weight was 124.53 kg. About a quarter of the patients had diabetes (26 %), dyslipidemia (27 %) and sleep apnea (25 %), while almost half had hypertension (47 %). The review reported on the three most common bariatric procedures currently performed by type of study, RCT or OBS. Average percent excess weight loss (%EWL) at 3 years was 57 % (RCTs) and 67 % (OBS). Remission of comorbid conditions occurred in the majority of patients: diabetes 92 % (RCT), 86 % (OBS); hypertension 75 % (RCT), 74 % (OBS); dyslipidemia 76 % (RCT), 68 % (OBS); sleep apnea 96 % (RCT), 90 % (OBS). Complication rates were 17 % (95 % CI 11–23 %) (RCT) and 10 % (95 % CI 7–13 %) (OBS) and reoperation rates were 7 % (95 % CI 3–12 %) (RCT) and 6 % (95 % CI 4–8 %) (OBS). In the RCT group the 30-day mortality rate was 0.08 % (95 % CI 0.01–0.24) compared to 0.31 % (95 % CI 0.01–0.75 %) for the OBS group.

### Mortality

Compared to non-surgical treatments for obesity, bariatric surgery is associated with a reduced risk of death [16, 18, 28–30]. A recent meta-analysis reported that bariatric surgery reduced the risk of mortality and cardiovascular mortality when compared to non-surgical interventions through a reduction in myocardial infarction, diabetes, and cancer-related deaths [28]. A reduced risk of mortality within 2–5 years of surgery has been reported in Canadian (89 %) [18], Australian (72 %) [30] and US (40 %) studies [29]. The Swedish Obesity Study (SOS) of more than 2000 patients, age and sex matched with obese controls, reported a 29 % reduction in mortality (adjusted HR 0.71 95 % CI 0.54–0.92) at 16 years [16]. More recent data from the SOS on 20 year mortality outcomes report an even more significant reduction (HR 0.47 95 % CI 0.29–0.76) in cardiovascular death (including MI and stroke) [31].

### Quality of life

Health related quality of life (HRQoL) encompasses measures of well-being and physical and psychosocial functioning [32]. Severe obesity is associated with significantly impaired HRQoL [32, 33]. In general, HRQoL improves in patients after bariatric surgery [19, 34–36]. However, findings are inconsistent [37], and improvements may often be limited to physical functioning with less improvement observed in emotional or mental functioning [36]. In addition very limited data on the long-term (>5 years) impact of bariatric surgery on quality of life exists [38].

### Complications

Data on complications suggest the benefits of bariatric surgery outweigh the harms [26, 27]. Perioperative mortality is low (<0.3 %) and declining [38]. The incidence of complications (e.g., pulmonary complications, vomiting, wound infection, hemorrhage, anastomotic leak) in the first 30–180 days after surgery varies widely from 4 to 25 % and depends on the definition of complication used, type of procedure, duration of follow-up, and individual patient characteristics. A study by Hutter et al. [39] reviewed the complication rates of 22,365 cases (944 LSG, 14,491 LRYGB, 988 open RYGB, 12,193 LAGB) and found that LSG fell below LRYGB but above LAGB for post-operative rates of morbidity, mortality, readmission, and reoperation rates. Long-term, the rates of reoperation as a result of complications, insufficient weight loss, or weight regain are a concern [40]. The effect of LSG on acid reflux is still controversial and requires further research [41]. Longer term there is some evidence that nutritional deficiencies (e.g., calcium, vitamin D, iron, zinc) may develop after bariatric surgery and the effect on bone health is unknown and also requires further study [26].

Emerging data from observational studies suggest that some bariatric procedures may increase the risk of substance misuse disorders (i.e., alcohol) and suicide [42]. Pharmacokinetic studies suggest that changes in the gastrointestinal anatomy after gastric bypass and sleeve gastrectomy may lead to a more rapid absorption of alcohol and increases in blood alcohol concentrations per dose, inadvertently increasing the frequency of physiological binges and subsequent alcohol misuse disorder [42, 43]. The risk of suicide after bariatric surgery may be increased but the cause is unclear [42]. The Utah Mortality study among others has demonstrated a small but significant increase in the number of suicides after surgery [29]. In addition, there are anecdotal reports of the development of addictive behaviors such as gambling, shoplifting, and driven sexual behavior [43]. It should be noted that these observations are limited due to the paucity of data on long-term psychological assessment in these patients.

### Economic evaluation

Studies have reported that bariatric surgery, mainly RYGB and AGB, are more cost-effective than non-surgical care [21, 22, 44–46] due to sustained weight reduction, decreased use of medications (especially for diabetes and cardiovascular disease), reduced outpatient and physician visits, and improved quality of life. However, the complexity of RYGB is associated with early complications resulting in increased inpatient stays. As well, the overall costs of AGB are increased due to reoperations and band removals [47]. Newer procedures, such as LSG have the potential to yield increased cost savings due to its relatively low complication rate and early evidence that clinical outcomes such as weight loss and comorbid resolution are comparable with RYGB [48]. There has not been an economic evaluation of LSG.

#### Laparoscopic sleeve gastrectomy as a treatment for severe obesity

LSG began as the first stage of a two-tiered operation of duodenal switch or RYGB for very large (e.g., BMI ≥ 50 kg/m^2^) or high-risk patients [3, 22, 49, 50]. Sleeve gastrectomy involves the surgical resection of the greater curvature and fundus of the stomach which creates an elongated tube or stomach ‘sleeve’ along the lesser curvature. This sleeve has a volume of 60–100 ml, effectively restricting caloric intake and increasing feelings of satiety. Removal of the fundus has also been associated with endocrine and metabolic effects [21], for example, the reduction of circulating levels of ghrelin – a hunger hormone, which may reduce the desire for food. It is not yet fully understood how LSG creates favorable metabolic changes and weight loss but this topic is a major focus of research. Since the early 2000’s, LSG grew in popularity as a stand-alone bariatric surgery because of the perceived technical ease of performing the surgery, associated obesity-related comorbidity improvement or resolution, and good short-term weight loss outcomes [25, 26]. It is now recognized as a primary bariatric surgery option and as a first-stage procedure in high-risk patients as part of a planned staged approach [26]. In a recent systematic review conducted by Victorzon in 2012, the author noted that the quantity, quality, and consistency of evidence for LSG for the treatment of obesity were low. Victorzon suggested that although numerous short-term studies had shown that LSG had good outcomes of between 45–60 % EWL, no longer term studies with >100 patients have been published [50]. For LSG, average clinically expected %EWL has been reported to be 56.1 % within the first year after surgery but there is limited data on sustained or continued weight loss longer term [51]. In a systematic review [51] the maximum weight loss reportedly occurred at 24 and 36 months following LSG surgery with %EWL’s of 64.3 % (46.1–75.0 %) and 66.0 % (60.0–77.5 %), respectively [51]. In a small study examining average %EWL 5 years following surgery %EWL was 86 % (50–103 %), but only 49 patients had complete follow-up data [52].

In general, bariatric surgery is effective in the resolution of many obesity-related medical comorbidities (e.g., type 2 diabetes mellitus [T2DM], hypertension, sleep apnea) [53], although data comparing the impact of RYGB and LSG on T2DM is inconsistent. A recent systematic review and meta-analysis compared the gold standard RYGB to LSG and found no significant difference in resolution of T2DM at 3 years (81 vs. 80 %) [54], however, other studies have shown inconsistent results when comparing the two procedures on improvements in glycemic control, achieving glycated hemoglobin value, and remission [49, 55, 56]. In contrast to T2DM, there does not appear to be a significant difference between RYGB and LSG on other comorbid conditions including dyslipidemia and hypertension [55, 57]. However, the development of reflux disease after LSG has been reported [57, 58] but a recent systematic review evaluating the effect of LSG on GERD reported inconsistent outcomes [59].

#### Knowledge gaps

The prevalence of severe obesity has increased dramatically in Canada and bariatric surgery is currently the only effective treatment option offered to medical refractory patients. Identifying cost-effective strategies to treat severe obesity must be a priority for governments and healthcare systems as healthcare expenditures of severely obese adults are double that of normal weight individuals [60]. Since 2003, there has been an exponential increase in the number of LSG’s performed as a treatment for severe obesity. There is evidence to support the efficacy of LSG in terms of weight loss and comorbid improvement/resolution, however data on other outcomes are limited (e.g., weight loss sustainability or recidivism, comorbid regression, nutritional deficiencies, non-alcoholic fatty liver disease [NAFLD], polycystic ovary syndrome [PCOS], quality of life, health services use and costs, short-term psychiatric problems), especially when based on a sample size of at least 80 % with follow-up of 2 years and beyond. In a review of bariatric literature by Puzziferri et al., to identify studies that had 80 % or greater follow-up at 2 years and beyond, < 3 % of studies (29/1136) met this inclusion criterion. Not all studies reported adequate weight loss data or outcome data on diabetes, hypertension, and dyslipidemia [61]. The authors state that the estimates of the treatment effect are very likely biased (overestimated) due to missing weight loss and other outcome data on representative samples. It is suggested that bariatric studies use more rigorous methods of patient engagement and retention in order to mitigate loss to follow-up [61].

#### The Newfoundland and Labrador Bariatric Surgery Cohort (BaSCo) study

##### Study objective

The objective of the current study is to examine short- to mid-term outcomes (2–4 years) associated with LSG. Eligible patients who have undergone LSG will be followed during and after surgery to assess health and other outcomes. Specific study objectives are to examine:

1. 30-day complication rate and mortality, and long-term complications (e.g., gastroesophageal reflux, micronutrient deficiencies).
2. reduction in weight and/or body mass index (BMI), including predictors of successful weight loss.
3. improvement or resolution of co‐morbid conditions (i.e. diabetes, hypertension, dyslipidemia, NAFLD, PCOS).
4. changes in prescription and over-the-counter medication use.
5. improvement in measures of health‐related quality of life and
6. the economic consequences associated with LSG (e.g., direct costs and indirect costs).

In the short- to mid-term we hypothesize a low rate of complications, a marked reduction in weight, improvement/resolution of comorbid conditions, a reduction in obesity-related medications, an improvement in quality of life, and a decrease in direct healthcare and indirect costs compared to pre-surgery costs.

## Methods/Design

### Study design

This is a prospective cohort study examining the effectiveness of LSG on patient health outcomes.

### Study population

#### Setting

The Provincial Bariatric Surgery Clinic is located in Eastern Health (EH). EH is the largest of four regional integrated health authorities in Newfoundland and Labrador (NL) and provides a full continuum of health services to a regional population of more than 300,000. It is also responsible for a number of unique provincial programs, for example, the Provincial Bariatric Surgery Clinic. The Provincial Bariatric Surgery Clinic was established in May 2011. This multidisciplinary team consists of three surgeons trained in bariatric surgery, a nurse practitioner, and other allied health professionals. Potential patients are most often referred via their primary care provider to the clinic and are invited to attend a pre-surgical education session. After the session, interested patients meet with the bariatric nurse practitioner where a detailed medical history is taken. Patients then meet with a bariatric surgeon, and if deemed to be a surgical candidate, they sign consent to undergo bariatric surgery, specifically LSG. At the time the team was developed, LSG was the only bariatric procedure offered to eligible patients in the province.

#### Patient selection criteria for bariatric surgery

The eligible population consists of all patients who (1) meet the Canadian Practice Guidelines criteria for the surgical treatment of obesity (BMI ≥35 with risk factors, or BMI ≥ 40) [3], (2) are referred by their primary care provider to the bariatric team using a standardized referral form submitted to a central intake system, and (3) receive preliminary eligibility screening by the nurse practitioner. Following mandatory attendance at a pre-surgical bariatric surgery general orientation and an education session provided either face-to-face or via webinar, patients are required to undergo an extensive pre-operative work-up which includes a 2-week diet trial (i.e.,1 week full-fluid diet and 1 week healthy eating) as well as a food journaling activity. All patients meet one-on-one or via Telehealth with the nurse practitioner for further assessment including a detailed review of their weight history and past weight loss attempts, blood work and a sleep study to identify and treat any sleep disordered breathing, as necessary. If any other medical concerns are identified, patients are consulted to the appropriate specialist (e.g., cardiologist, endocrinologist, respirologist) based on their comorbid condition. An appointment with one of the three bariatric surgeons in the bariatric surgery clinic is arranged to obtain formal surgical consent.

#### Inclusion criteria

- Male or female patients between 19 and 70 years of age
- BMI ≥ 40 kg/m^2^ or ≥ 35 kg/m^2^ with significant obesity-related comorbidities
- Maximum BMI 60 kg/m^2^ as per NL Bariatric Surgery Program guidelines
- Attempted nonsurgical weight loss in the past
- Deemed medically, psychologically, and emotionally stable to consent to surgery and partake in a diet and lifestyle modification regime

#### Exclusion criteria

- Pregnant

#### Recruitment for bariatric surgery study

**Fig. 1:**
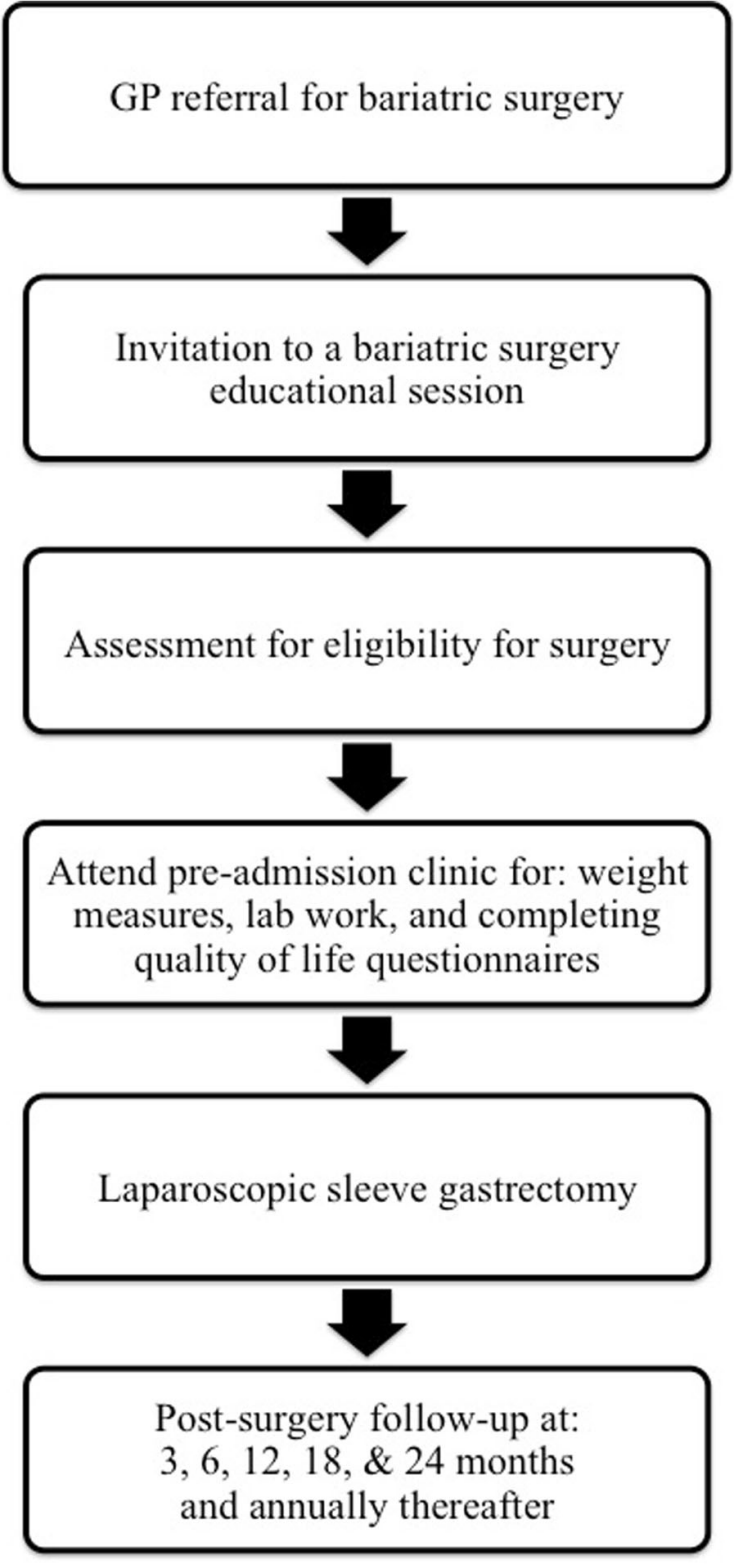
The NL Bariatric Surgery Cohort Study: Patient Flow

Once deemed eligible for surgery, a research nurse coordinator approaches every patient to discuss potential interest in the study. The research nurse gives each eligible participant a brief explanation of the study, an introductory letter addressed from the researcher, an information sheet, and consent form, and asks if she can contact them to discuss the research project in greater detail after the potential participant has had 24 to 48 h to review the materials. If agreeable to this request, the research nurse calls or makes contact with the potential participant at the bariatric surgery clinic or by telephone to answer any questions and set-up a time during the next visit to the clinic to obtain written consent and complete baseline data collection. The study protocol is illustrated in Fig. 1.

#### Surgical intervention

The surgical intervention is performed laparoscopically under general anesthesia. A 6 port technique is performed. The vascular supply of the stomach is divided along the greater curve, starting 5 cm proximal to the pylorus and up to the angel of His. A gastric sleeve is created using multiple applications of a 60 mm linear stapler after the sleeve size is calibrated using a 42Fr bougie. The gastric specimen is removed via the left upper quadrant port site. An endoscopic air leak test is routinely done to confirm an intact staple line, and an upper gastrointestinal contrast study is performed on the first postoperative day before the introduction of oral liquid diet. If this is clear, patients are started on a clear liquid diet and are normally discharged home on post-operative day two with dietary instructions.

### Data collection

Patients are followed by the provincial bariatric surgery clinic team as standard of care. The patients are assessed by a bariatric surgeon 6–8 weeks post-surgery. In‐person follow-up visits or assessments via Tele-health with the Nurse Practitioner/bariatric care team take place at 1, 3, 6, 12, 18, and 24 months, and annually thereafter.

### Study measures

Standardized case report forms (SCRFs) developed in collaboration with Eastern Health and in consultation with other Canadian bariatric programs are used to collect relevant research and clinical data. Clinical data such as weight measures and blood work are collected by the clinical team (the nurse practitioner) and provided to the research team via a research nurse. The research nurse collects data on individual patients at each follow‐up visit using the SCRFs (Table 1).

**Table 1:** Study measures and data collection time periods

Study Measures	Source	Variable	Time frame (s)
Socio-demographics/Lifestyle	Patient Interview	Age, sex, ethnicity, current marital status, highest level of education, occupation, current employment status, current household income level, smoking status (current, past, never)	Pre-surgery
Anthropometric	Standardized Case Report Form	Weight^1^, height^2^, BMI^3^	Pre-surgery, 3, 6, 12, 18, 24 months and annually thereafter
Medical History/Self-reported baseline comorbid conditions	Standardized Case Report Form	Diabetes, hypertension, dyslipidemia, sleep apnea, NAFLD, PCOS, etc.	Pre-surgery
Assessment of obesity-related comorbid conditions to evaluate changes in comorbid status	Standardized Case Report Form Patient Interview	Diabetes, hypertension, dyslipidemia, NAFLD, PCOS	3, 6, 12, 18, 24 months and annually thereafter PCOS will be assessed at 12 months only
Medication use	Standardized Case Report Form	Prescribed and over-the-counter medications (name, type, dosage, frequency and duration)	Pre-surgery, 3, 6, 12, 18, 24 months and annually thereafter
Clinical characteristics	Standardized Case Report Form	Blood pressure^4^, heart rate	Pre-surgery, 3, 6, 12, 18, 24 months and annually thereafter
Laboratory values	Meditech laboratory module	HbA1c, fasting blood glucose, insulin level, creatinine, fasting lipid panel (including total cholesterol, LDL, HDL, triglycerides), high sensitivity (hs) CRP, GGT, albumin, total bilirubin, ALP, ALT, total protein, ferritin, hemoglobin, MCV, TSH, 25-OH D, Vitamin B12	Pre-surgery, 3, 6, 12, 18, 24 months and annually thereafter
Health-related QoL	Validated surveys	SF 12v2, EQ‐5D, IWQoL‐Lite	Pre-surgery, 6, 12, 18, 24 months
Operative data	Clinical database and chart review	Minor and major complications	During surgery and up to 30 days post-surgery

^1^Body weight is measured using a validated, calibrated bariatric scale and recorded to the nearest 0.1 kg, with the subject wearing light indoor clothing with empty pockets, no shoes, and an empty bladder

^2^Height is measured to the nearest 0.1 cm using a wall-mounted stadiometer

^3^BMI = weight in kg/height in m^2^

^4^A single reading taking using an automated blood pressure monitor and using an appropriately sized blood pressure cuff will be recorded with the subject seated in a chair and after 5 min of rest

### Evaluation of health care utilization and costs

The impact of bariatric surgery on the health system will be examined by comparing health care use and costs (e.g., physician visits, hospitalizations, medications and associated costs) 3 years pre‐and 2 years post‐surgery (Table 2). In NL, the Centre for Health Information (NLCHI) maintains a number of key health-related data sources on behalf of the provincial Ministry of Health. These databases comprehensively capture patient-specific health care resource utilization. These include vital statistics (mortality) and validated, high quality, detailed measurement of the following health care resources and costs: inpatient and outpatient encounters, physician billings, diagnostic tests, procedures, and long-term care use. In addition to providing detailed information allowing patient-specific costing of each encounter, inpatient databases record diagnostic and procedural information using the ICD-10-CA and CCI classification systems [62]. Using unique provincial health insurance numbers, the NLCHI will link the bariatric clinical/research data with administrative health services data and provide the research team with a de‐identified database of health services use and costs for data analysis purposes [63, 64].

**Table 2:** Short to mid-term health care use and costs in surgical patients

Cost Category	Units of Resources	Newfoundland & Labrador Source Details	Newfoundland & Labrador Costing Source	Time Frame
1. Inpatient encounters	# of hospitalizations (acute care) Length-of-stay (LOS)	Newfoundland and Labrador Centre for Health Information (NLCHI) Clinical Database Management System (CDMS), based on the Discharge Abstract Database (DAD) includes LOS, procedures performed, associated diagnoses, RIW and complexity values	NLCHI/Canadian Institute for Health Information (CIHI) methodology to cost acute care encounters using resource-intensity-weights (RIW) and complexity values	Provided by fiscal year. Referenced to time zero (date of surgery)
2. Outpatient encounters	# encounters # procedures	NLCHI CDMS (based on DAD) includes surgical day care procedures	NLCHI/CIHI methodology to cost surgical day care encounters	Provided by fiscal year. Referenced to time zero
3. Physician Fees (Fee for Service)	# of encounters Provider specialty Service provided	Medical Care Plan (MCP) Physician Claims	MCP Physician Claims	Provided by fiscal year. Referenced to time zero
4. Physician Fees (self-report)	# of encounters Provider specialty Service provided	Patient interview (standardized case report form)	Estimated using MCP Physician Claims	Information obtained every 6 months
5. Medications	Name dosage, frequency & duration	Patient interview (standardized case report form)	NL Drug (Provincial Formulary)	Information obtained every 6 months
6. Transfer Payments	Unemployment insurance Disability benefits	Patient interview (standardized case report form)	Patient Reported	Information obtained every 6 months
8. Employment status, absenteeism	Employment status in past year (# hours/week, # weeks) Absenteeism in past year (# days) Annual income (by quintile)	Patient interview (standardized case report form)	Average wage rate by age, sex and region from Statistics Canada	Information obtained every 6 months
9. Weight Loss Interventions	Weight loss program, meal replacements, physical trainer, exercise programs, alternative therapies (Binary Y/N)	Patient interview (standardized case report form)	Patient reported out of pocket cost Costs lists of rehabilitation supplies & equipment	Information obtained every 6 months
10. Mobility and Medical	Mobility aids, home modification/renovations, rehabilitation, paid personal assistance (household activities and home productivity, driving) (Binary Y/N)	Patient interview (standardized case report form)	Patient reported out of pocket cost/co-payments for medical services Costs lists of rehabilitation supplies & equipment	Information obtained every 6 months

*DAD* Discharge Abstract Database, *LOS* Length of stay, *CIHI* Canadian Institute for Health Information, *CDMS* Clinical Database Management System, *RIW* Resource Intensity Weight, *MCP* Newfoundland and Labrador Medical Care Plan, *EH* Eastern Health

### Outcomes

#### Primary outcome: weight loss

Absolute (kg, BMI) changes and percent changes (%EWL, %TWL, % change in BMI) from baseline will be reported. Percent Excess Weight Loss (%EWL) is calculated as100% x ([W0 – W1]/EW0) where W0 is the weight (kg) at the time of the surgery, W1 is the weight (kg) at the last follow‐up and EW0 is the excess weight at the time of the surgery, based on ideal weights found in the Metropolitan tables for middle frame individuals [65]. Percent Total Weight Loss (%TWL) is calculated as 100 % x ([W0-W1]/W0) where W0 is the weight (kg) at the time of the surgery, W1 is the weight (kg) at the last follow‐up. Using definitions of successful weight loss 12 months after bariatric surgery, we will calculate the proportion of patients who attain (>50 % EWL). In addition, the proportion of subjects that achieve clinically important weight loss (i.e. ≥ 5 and 10 %) will be reported [3].

#### Sample size calculation

The primary outcome for the sample size determination was defined as successful weight loss at 12 months after bariatric surgery. This was calculated as percentage of patients who attain (>50 % EWL) at 12 months post-surgery. Sample size was estimated using an online calculator for population surveys as follows: *n* = (Z^2^ × P (1 – P))/E^2^ where Z = value from standard normal distribution corresponding to desired confidence level (Z = 1.28 for 80 % CI), P is expected true proportion and E is desired precision (half desired CI width). Assuming that 50–60 % of patients undergoing LSG will achieve the primary outcome of weight loss (>50 % EWL), a total of 165 patients will be required for a precision of ±5 % at a confidence level of 80 %. To account for missing information from some patients, the sample size has been inflated to 200.

#### Secondary outcomes

1. Complications of surgery
  30-day complication rate and mortality will be reported. Longer-term complications such as gastroesophageal reflux and micronutrient deficiencies will be examined. To examine micronutrient deficiencies, the following biochemical parameters associated with micronutrients will be assessed (25-hydroxyvitamin D (25-OH-D), parathyroid hormone (PTH), calcium, vitamin B12, ferritin, mean cell volume (MCV) and hemoglobin). The analysis for 25-OH-D used liquid chromatography coupled with tandem mass spectrometry. Hemoglobin and MCV were analyzed on the Beckman Coulter®HMX Hematology Analyzer, while ferritin, vitamin B12, and intact PTH were analyzed on the Architect i System by Abbots Diagnostics. Calcium was analyzed on the Architect c System by Abbots Diagnostics.
2. Comorbid Condition Improvement/Resolution
  Improvement in comorbid conditions (i.e. hypertension and dyslipidemia) is defined as any reduction in medication use or treatment. Comorbidity resolution is defined as complete cessation of treatment/medication.
  For the comorbid conditions of prediabetes and type 2 diabetes mellitus, specific definitions of diagnosis are used as outlined in the Canadian Diabetes Association Clinical Practice Guidelines [66]. Prediabetes is defined as fasting plasma glucose (FPG) levels of 6.1–6.9 mmol/L and/or glycated hemoglobin (A1C) levels of 6.0–6.4 % [66]. Type 2 diabetes mellitus is defined as FPG ≥ 7.0 mmol/L and/or A1C ≥ 6.5 % [66].
  Additional criteria will used to assess improvement and resolution of prediabetes and diabetes [67]. Case definitions of improvement and remission of prediabetes will be as follows: *Improvement* will be based on achievement of the following: (1) lower glycemic measures (2) at least 1 year’s duration, and (3) pharmacologic therapy required but at a lower dose. *Normalization* is defined as (1) normal glycemic measures (2) at least 1 year’s duration, and (3) pharmacologic therapy required but at a lower dose. *Remission* is based on (1) normal glycemic measures (2) at least 1 year’s duration, and (3) no active pharmacologic therapy. Case definitions of improvement and remission of Type 2 diabetes mellitus [67] will be defined as follows. *Improvement* will be based (1) hyperglycemia below diagnostic thresholds for T2DM (2) at least 1 year’s duration, and (3) pharmacologic therapy required but at a lower dose. *Partial remission* is based on the following (1) hyperglycemia below diagnostic thresholds for T2DM (2) at least 1 year’s duration, and (3) no active pharmacologic therapy. *Complete remission* is based on the following criteria (1) normal glycemic measures (2) at least 1 year’s duration, and (3) no active pharmacologic therapy.
  The status of specific biochemical parameters associated with NAFLD prior to and following LSG will be assessed using the following markers: total bilirubin, alanine phosphatase (ALP), alanine transaminase (ALT), gamma glutamyl transpeptidase (GGT), high-density lipoprotein (HDL), low-density lipoprotein (LDL), and triglycerides. Biochemical parameters associated with NAFLD were analyzed on the Architect c System by Abbots Diagnostics.
  The prevalence of PCOS will be determined prior to surgery based on self-report while changes in related (e.g., period frequency and regularity, menstrual flow and intensity, hirsutism, acne, fertility, post-surgery conception) and metabolic symptoms (insulin sensitivity, T2DM, dyslipidemia, hypertension) will be examined 1-year after LSG.
3. Change in medication use
  Changes in prescription and over-the-counter medication use and associated costs will be examined including total number of medications and changes (dose changes, medication discontinuations, and initiation of additional medications) by disease indication.
4. Quality of Life
  Health related quality of life is assessed using validated instruments. Generic and disease-specific instruments will be used. All participants will complete the Short Form (SF)-12, Euroqol (EQ)-5D, and the Impact of Weight on Quality of Life (IWQOL)-Lite pre-surgery at 6, 12, 18, and 24 months after surgery. The SF-12 (Version 2) is a condensed 12-question version of the SF-36 [68]. It yields a physical and a mental health component summary score. Three-to-five point difference in either score are considered clinically meaningful [69, 70]. The EQ-5D is an indirect preference-based health survey that consists of a 5 dimension descriptive system [71] and an overall health visual analog scale (EQ- VAS). A 0.03 point difference in EQ-5D index score and 10 point difference on the EQ-VAS are considered clinically meaningful [72]. The IWQOL-Lite is a short form of the IWQOL and is the first instrument specifically developed to assess the effects of obesity on the quality of life of persons who are seeking weight loss treatment [73]. The IWQOL-lite consists of 31 items that describe 5 domains of obesity-specific HRQL. A difference in the total score of 7–12 points for IWQOL-Lite is considered clinically meaningful [74].
5. Economic consequences of bariatric surgery
  Direct healthcare use/costs (e.g., hospitalizations, physician visits, medication use) and indirect costs (e.g., cost of weight loss interventions, employment and absenteeism, home productivity, transfer payments) will be compared 3 years prior to surgery and 2 years post- surgery in order to determine the economic consequences of bariatric surgery.

### Data analysis

Non-identifying patient information obtained from the standardized case report forms used to collect patient information at baseline, administrative records and medical chart reviews were entered into a Statistical Package for Social Sciences database. All statistical analyses will be performed using IBM SPSS Statistics for Windows, version 20.0 [75]. Descriptive analysis will include demographic details of patients pre-surgery and clinical information collected at the time of surgery. In each analysis to be conducted to address the six main research objectives for this program, categorical variables (such as 30-day complication rate and mortality; and long-term complications e.g., gastro esophageal reflux, micronutrient deficiencies) will be presented as frequencies and chi-square tests conducted to identify differences in pre–post surgical outcomes. Continuous variables with a normal distribution will be reported as means ± SD. Variables with a non-normal distribution will be reported as medians and interquartile ranges. For example, health services use such as number of physician visits often has a non‐normal distribution; therefore median and quartiles will be reported when comparing use pre‐ and post‐surgery. The Analysis of Variance (ANOVA) or the paired t-method will be used to assess differences in continuous outcome pre and post-surgery. Pairwise-comparison of pre with post-surgical patients’ outcomes at 3, 6, 12, 18 and 24 months will be done using matched t-tests for continuous outcomes such as BMI. Regression analyses methods will be performed to identify independent predictors of outcomes of interest (i.e., reduction in weight and/or BMI, including predictors of successful weight loss; improvement or resolution of co‐morbid conditions (i.e., diabetes, hypertension, dyslipidemia, NAFLD, PCOS); changes in prescription and over-the-counter medication use; improvement in measures of health‐related quality of life. For continues outcomes, mean differences, standard errors, 95 % confidence intervals and p-values will be presented. For categorical outcomes, odds ratios and 95 % confidence intervals will be presented. Generalized estimating equations with binary logistic regression will be used for all response or outcome events to take into account the correlations between repeated measures post-surgery providing an estimated adjusted odds ratio and 95 % confidence intervals. Statistical significance is set at *p* < 0.05.

To examine the predictors of change in patient’s 2 years post-surgery, MIXED modelling regression approach [76, 77] implemented in SPSS will be used to assess baseline patient’s factors that determine individual changes in individual maintaining weight loss over the 24 months. The mixed modeling approach will also allow us to take into account the correlations between repeated measures and accounts for missing data over time. Statistical significance effect of factors producing changes or reduction in weight and/or BMI, including predictors of successful weight loss overtime in the 24 month post-surgery would be set at *p* < 0.05. Cost-effectiveness will be performed to evaluate the economic consequences associated with LSG. The economic analyses will be carried out on a per protocol basis and would be based on patients for whom there was complete resource use and health outcome data provided by the NL Centre for Health Information.

### Ethics and privacy

We have received full ethics approval for the NL Bariatric Surgery Study [Health Research Ethics Authority (HREA) 11.101]. Patient informed consent was obtained and subjects were informed they could withdraw from the study at any time. The research nurse is the only member of the team who has access to identifying patient information. This information is kept in a locked cabinet in a secure office. De-identified data is secured in password protected files and only accessed by researchers involved in the study for the purpose of data analysis. As with previous projects involving the compilation of sensitive datasets from multiple sources such as cancer registries and physician billings, the protection of individual privacy and anonymity and confidentiality will be maintained by having NLCHI act as a trusted third party.

## Discussion

In summary, the demand for LSG as a stand-alone procedure is growing in popularity as a treatment for class II and III obesity. In a joint report “Developing a Research Agenda to Support Bariatric Care” published by the CIHR and the Canadian Obesity Network in 2010, the gaps in research related to bariatric surgery were highlighted [78]. Although LSG was not specifically highlighted in the report, compared to other bariatric surgeries, research on LSG as a treatment for severe obesity is limited due to its relatively recent provision as a stand-alone procedure. The start-up of a bariatric surgery program offering LSG as a treatment for severe obesity combined with the limited research on LSG provided an opportunity for research. Consequently, an Integrated Knowledge Translation Team was established at Memorial University, comprised of academic researchers, healthcare professionals, decision-makers, and policy makers, and a program of research on bariatric care was developed to address key gaps in the research literature. One of the studies designed and implemented within this program of research and presented in this paper is a quasi-experimental study that aims to address current knowledge gaps in LSG by generating prospective, population-based Canadian outcome data. This study is being conducted in a provincial bariatric surgery program within a tertiary care teaching centre and the results will be generalizable to similar programs offering bariatric surgery. A current review continues to highlight a major concern previously identified by others [79, 80] associated with bariatric surgery as a treatment for obesity. It is not the magnitude of initial weight loss which has been well documented but the mid- to long-term sustainability of weight loss that is under question. Very few bariatric studies report long-term results with sufficient patient follow-up (>80 %) in order to minimize biased results. In addition, insufficient evidence exists regarding long-term outcomes for LSG [61]. In response to this concern and as part of the current study protocol, a clinical database to house all program data in order to monitor longer-term (>5 years) patient health outcomes has been developed which will also allow for data linkages with administrative data to examine health care use and costs in the future. Further interventions being used in order to decrease attrition and increase patient engagement include the use of technology (e.g., Tele-health and webinar sessions for educational purposes). As of August 2015, 100 % recruitment has been achieved with completion of follow-up to be completed by September 2016 with study results to be published in late 2016 and early 2017. Our study population is 82 % female, with an average age, weight, and BMI of 44 years, 135 kgs and 49 kg/m^2^, respectively. The prevalence rates of hypertension and dyslipidemia were approximately 50 and 43 % had T2DM. Our study participants are similar to other patient populations that are eligible for and undergoing bariatric surgery elsewhere in Canada [15, 21], increasing the external validity of our study findings.

## Abbreviations

25-OH-D: 25 Hydroxy Vitamin D
A1C: Glycated hemoglobin
AGB: Adjustable gastric banding
ALP: Alkaline phosphatase
ALT: Alanine transaminase
ANOVA: Analysis of variance
BMI: Body mass index
BPD/DS: Biliopancreatic diversion/duodenal switch
EQ-5D: Euroqol 5D
EWL: Excess weight loss
FPG: Fasting plasma glucose
GGT: Gamma-glutamyl transferase
HDL: High-density lipoprotein
HREA: Health Research Ethics Authority
HRQoL: Health related quality of life
IWQoL: Impact of weight on quality of life
LAGB: Laparoscopic adjustable gastric banding
LDL: Low-density lipoprotein
LSG: Laparoscopic sleeve gastrectomy
MCV: Mean cell volume
NAFLD: Nonalcoholic fatty liver disease
NL: Newfoundland and Labrador
NLCHI: Newfoundland and Labrador Centre for Health Information
OBS: Observational study
PCOS: Polycystic ovarian syndrome
PTH: Parathyroid hormone
RCT: Randomized controlled trial
RYGB: Roux-en-y gastric bypass
SCRFs: Standardized case report forms
SD: Standard deviation
SF-12: Short form 12
SF-36: Short form 36
T2DM: Type 2 diabetes mellitus
VAS: Visual analogue scale
